# LoxP-FRT Trap (LOFT): a simple and flexible system for conventional and reversible gene targeting

**DOI:** 10.1186/1741-7007-10-96

**Published:** 2012-11-30

**Authors:** Barbara H Chaiyachati, Ravinder K Kaundal, Jiugang Zhao, Jie Wu, Richard Flavell, Tian Chi

**Affiliations:** 1 Department of Immunobiology, Yale University Medical School, 300 Cedar Street, New Haven, CT 06520, USA; 2 Key laboratory of Pig Industry Sciences, Ministry of Agriculture, Chongqing Academy of Animal Sciences, Rongchang 402460, Chongqing, China

## Abstract

**Background:**

Conditional gene knockout (cKO) mediated by the Cre/LoxP system is indispensable for exploring gene functions in mice. However, a major limitation of this method is that gene KO is not reversible. A number of methods have been developed to overcome this, but each method has its own limitations.

**Results:**

We describe a simple method we have named LOFT [LoxP-flippase (FLP) recognition target (FRT) Trap], which is capable of reversible cKO and free of the limitations associated with existing techniques. This method involves two alleles of a target gene: a standard floxed allele, and a multi-functional allele bearing an FRT-flanked gene-trap cassette, which inactivates the target gene while reporting its expression with green fluorescent protein (GFP); the trapped allele is thus a null and GFP reporter by default, but is convertible into a wild-type allele. The floxed and trapped alleles can typically be generated using a single construct bearing a gene-trap cassette doubly flanked by LoxP and FRT sites, and can be used independently to achieve conditional and constitutive gene KO, respectively. More importantly, in mice bearing both alleles and also expressing the Cre and FLP recombinases, sequential function of the two enzymes should lead to deletion of the target gene, followed by restoration of its expression, thus achieving reversible cKO. LOFT should be generally applicable to mouse genes, including the growing numbers of genes already floxed; in the latter case, only the trapped alleles need to be generated to confer reversibility to the pre-existing cKO models. LOFT has other applications, including the creation and reversal of hypomorphic mutations. In this study we proved the principle of LOFT in the context of T-cell development, at a hypomorphic allele of *Baf57/Smarce1 *encoding a subunit of the chromatin-remodeling Brg/Brahma-associated factor (BAF) complex. Interestingly, the FLP used in the current work caused efficient reversal in peripheral T cells but not thymocytes, which is advantageous for studying developmental epigenetic programming of T-cell functions, a fundamental issue in immunology.

**Conclusions:**

LOFT combines well-established basic genetic methods into a simple and reliable method for reversible gene targeting, with the flexibility of achieving traditional constitutive and conditional KO.

## Background

Conventional gene knockout (KO) technologies such as LoxP/Cre-mediated conditional gene KO (cKO) are widely used for discovering gene functions. A key limitation of these methods is that the KO is irreversible. It is therefore impossible to determine if, for example, the malignancies and neurological disorders reported in *p53 *and *MeCP2 *KO mice, respectively, can be cured by restoring gene functions, a question of obvious clinical relevance. Because the KO in the original mouse models is not reversible, special strains have to be generated to address these questions, which entails substantial amounts of work [1-3]. Reversible KO would also be invaluable for studying epigenetic programming, a central issue in developmental biology. Specifically, during lineage development, transient action of environmental cues is thought to irreversibly modify (or 'program') the epigenetic states of target genes in the developing cells, such that the altered epigenetic states can persist and be propagated to mature progeny cells without the continuous presence of the initiating cues [4]. Defining the role of a gene in developmental programming requires deleting the gene in immature cells and analyzing the resultant defects in mature cells, but the gene controlling developmental programming may also be expressed and functioning in mature cells, which complicates data interpretation, given that conventional KO strategy is not reversible. For example, deleting the chromatin-remodeling factor Mi-2b in immature T cells impairs proliferation of mature T cells [5], but because Mi-2b is expressed not only in immature but also in mature T cells, it is unclear if the proliferation defect reflects a developmental role of Mi-2b. The only way to directly address such an issue is to eliminate the protein in immature cells. and then restore its expression in mature cells.

Multiple methods have been devised to achieve reversible gene regulation, but each has limitations. In one method, endogenous genes are modified so that their expression is now driven by tetracycline-regulated artificial transcription activators expressed from the endogenous regulatory elements, thus allowing for reversible gene regulation, but it is difficult to recapitulate the expression levels of the endogenous genes with the synthetic activators [6-9]. In an alternative method, tetracycline-controlled transcriptional silencer (tTS), a tetracycline-regulated transcription repressor, has been successfully used to reversibly inhibit the expression of *Hoxa2 *and *Htr1a*, but whether this method is generally applicable to other genes remains unclear, and furthermore, the only tTS transgenic line currently available expresses tTS in various tissues, and is hence unsuitable for tissue-specific inhibition. Regulated expression of small hairpin RNA has also been used for reversible gene repression, but the repression is usually incomplete [2]. Finally, transcription stop sequences or gene-trap cassettes, which are removable/inactivable, can be inserted into target genes, leading to constitutive KO that can be conditionally rescued, but this strategy is not suitable for conditional induction of gene KO [1,3,10,11].

In this paper, we describe a straightforward and robust method for reversible cKO without these limitations. The method, which we dub LOFT [LoxP-flippase (FLP) recognition target (FRT) Trap], combines cKO with gene trapping, a well-established method for insertional mutagenesis [12-16]. In its simplest form, a gene-trap cassette consists of a promoterless selectable marker flanked by a splice acceptor (SA) and a polyadenylation (pA) sequence. When inserted into an intron of an expressed gene, the SA captures the upstream exon while the pA sequence truncates the transcript, thus producing a fusion protein between the N-terminus of the trapped protein and the selectable marker. Thus, gene traps simultaneously inactivate and report the expression of the trapped gene. Gene trapping can be made conditional by flanking gene-trap modules with LoxP/FRT sites [10,11,17]. LOFT combines Cre-catalyzed cKO with FLP-catalyzed reversible trapping to achieve reversible cKO. LOFT can also be used to create conventional KO mice. We report a proof-of-concept study using the gene encoding Brg/Brahma-associated factor (BAF)57, a subunit of the chromatin-remodeling BAF complex.

The BAF complex, a prototypical mammalian ATP-dependent chromatin remodeler complex (CRC), is widely expressed, and plays diverse, often tissue-specific roles in gene regulation [18-20]. Although called ATP-dependent CRC, the complex can also regulate target genes without using the classic ATP-dependent chromatin-remodeling activity [21]. Indeed, although the complex consists of more than ten subunits, a group of four core subunits, including the catalytic subunit Brahma-related gene (BRG)1, is fully sufficient to reconstitute ATP-dependent chromatin-remodeling *in vitro *[22]. The functions of the remaining accessory subunits are poorly understood, but may contribute to the ATP-independent functions of the BAF complex and/or modulate the classic remodeling activity of the BAF complex. The 57 kDa high mobility group (HMG) protein BAF57 (also known as SMARCE1; Switch/sucrose non-fermentable (SWI/SWF) related matrix-associated actin-dependent regulator of chromatin subfamily E member 1) is the first known accessory subunit [23]. BAF57 is important for T -cell development in mice [24], and for regulating apoptosis [25], the cell cycle [26] and functions of the androgen and estrogen receptors [27-29] in tumor lines. Furthermore, BAF57 is strongly expressed in human endometrial carcinoma, and serves as a marker of poor prognosis [30].

We are interested in further studying the roles of BAF57 in T- cell development in the thymus, which is arguably the best-defined ontogenetic system in vertebrates [31]. The earliest thymocytes are double-negative (DN) cells lacking the antigen coreceptor CD4 or CD8. These cells undergo extensive proliferation, and express both CD4 and CD8 to become double-positive (DP) cells. DP cells bifurcate into CD4 helper and CD8 cytotoxic cells, the two major subsets of T lymphocytes in the adaptive immune system, which are marked by CD4 and CD8 expression, respectively. We previously explored the role of BAF57 in T cells using a BAF57 dominant-negative mutant. BAF57 is a protein of 411 amino acids (aa) consisting of several conserved domains, including the N-terminal proline-rich domain (23 aa) with unknown functions, the HMG domain (aa 66 to 133) that binds DNA, a domain rich in Asp, His, Leu and Ile (NHRKI), and the C-terminal domain rich in acidic residues [32]. The functions of these domains are unknown except for the DNA-binding of the HMG domain [23]. We found that thymocyte-specific expression of a dominant-negative mutant of BAF57 lacking the N-terminal 133 aa including the HMG causes reciprocal CD4/CD8 misregulation during T-cell development, but the mutant does not significantly impair production or function of mature T cells [24,33]. Because the dominant-negative mutation impairs only a specific aspect of BAF57 function, the roles of BAF57 in T cells remain incompletely understood. In particular, it is unclear if BAF57, acting in thymocytes, can epigenetically program the function of mature T cells. This problem motivated us to develop the reversible cKO method LOFT.

## Results

### LOFT: Basic rationale

**Figure 1:**
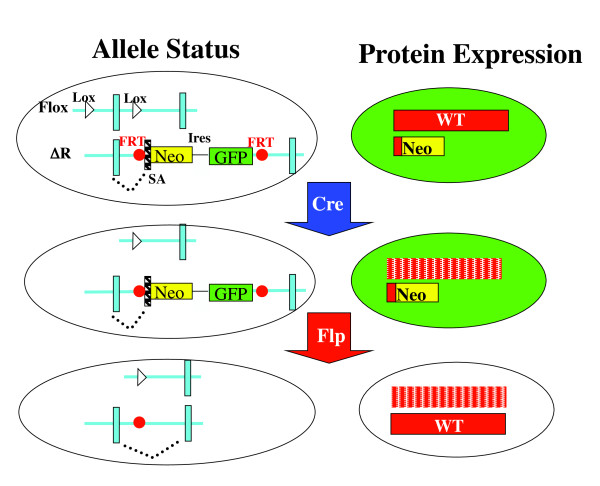
**Strategy for green fluorescent protein (GFP)-labeled reversible conditional gene knockout (cKO)**. This method requires a conventional floxed allele (Flox) paired with a multi-functional reversible KO (ΔR) allele (top left), and sequential action of Cre and Flp recombinases (middle and bottom). Depicted are the status of the alleles (left) and the corresponding protein expression patterns (right). SA, splicing acceptor; Neo, *neomycin resistance gene*; FRT, Flippase recognition target (red dots).

cKO followed by conditional restoration of gene expression is achieved with a pair of alleles of a target gene: a floxed allele and a reversibly trapped allele that is a null by default but can be conditionally converted to a wild-type (WT) allele. The latter allele is designated ΔR, where R denotes 'reversible' (Figure 1). The key component of the ΔR allele is a gene-trap cassette consisting of the neomycin phosphotransferase (Neo) and an Ires-green fluorescent protein (GFP) construct. This cassette is inserted into an intron, thus capturing the upstream exon to produce a fusion protein between the N-terminus of the target protein and the neomycin phosphotransferase, the former moiety being inactive, and the latter serving as the selection marker for successfully targeted embryonic stem (ES) cells if the target gene is expressed in those cells. In addition, GFP is co-expressed with the fusion protein, which reports the expression pattern of the target gene. Importantly, the gene-trapping cassette is flanked by FLP recombination target (FRT) sites, allowing for conditional excision of the cassette in the presence of active FLP. The removal of the gene-trapping cassette restores the expression of full-length protein, concomitant with the loss of GFP expression. The floxed and ΔR alleles can be used separately to achieve conditional and constitutive gene KO, respectively, but in combination, they allow for reversible cKO: in mice bearing both alleles and also expressing conditional Cre and FLP, sequential function of the two enzymes will lead to target gene knockout followed by restoration of its expression, and this process can be monitored by the loss of GFP expression (Figure 1). The construct depicted in Figure 1 is not suitable for genes not expressed in ES cells. In such cases, one may delete the Neo-Ires sequence in the vector and insert a Neo expression cassette (containing a promoter active in the ES cells) between GFP-pA sequence and the 3' FRT, thus turning the promoter-trapping vector into a simple conventional knock-in construct. Of note, although the major application of LOFT is likely to be reversible cKO, variations of the theme are readily conceivable. For example, if excision of the floxed sequence leads to the production of a deletion mutant rather than to elimination of the entire protein, then LOFT may be used to reverse the effects of such hypomorphic mutations, as described below.

### Generation of *Baf57^F ^*and *Baf57^ΔR^*

**Figure 2:**
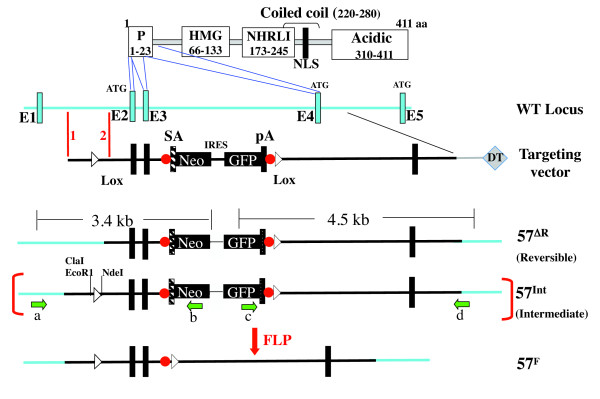
***Baf57 *allelic series produced by a single gene-trap vector**. The domain structure of BAF57 is depicted at the top, where the N-terminal 23-residue proline-rich region (P) is encoded by Exons 2 to 4. BAF57 translation normally starts at the ATG in exon 2, but exon 4 (and exon 5) also contains an ATG that is in frame with the remaining sequence. The targeting vector contains a pair of Flippase recognition target (FRT) sites (red dots) and a pair of LoxP sites (triangles) flanking the gene-trap cassette. During homologous recombination, crossing over at the left arm can happen downstream or upstream of the 5' Lox P site (red vertical lines 1 and 2 flanking the 5' Lox P site), generating *Baf57^ΔR ^*and the intermediate *Baf57^Int ^*alleles, respectively, the latter convertible to *Baf57^F^*, where exons 2 and 3 are floxed. DT, diphtheria toxin expression cassette; HMG, high mobility group; NHRLI, a domain rich in Asp, His, Leu and Ile; NLS, nuclear localization signal. Green arrows denote PCR primers used in Figure 3A.

Our goal was to study the potential roles of BAF57 in developmental programming of T cells, which required deleting BAF57 in immature T cells, followed by the reversal of the KO in mature T cells. Because our previous studies involving BRG1 suggests that complete elimination of BAF57 might block T-cell development and make the experiment unfeasible [21,34], we sought to reduce rather than eliminate BAF57 expression. To this end, we floxed exons 2 and 3, which encode the first 18 residues of BAF57, and named the resulting allele BAF57^F ^(Figure 2). Deleting the two exons would be expected to cause expression of a truncated BAF57 protein that starts with a methionine encoded by an ATG in exon 4. Because the ATG in exon 4 is not embedded in the Kozak consensus sequence, the mutant should be expressed at lower levels than the WT. In addition, the residual protein might not be fully active, given that it lacks the bulk of the conserved proline-rich domain. Thus, we would expect *Baf57^F ^*to be a conditional hypomorphic allele.

*Baf57^F ^*and *Baf57^Δ^*^R ^were generated with a single targeting vector by exploiting crossing-over site variability during homologous recombination (Figure 2) [35-37]. To facilitate the generation of *Baf57^Δ^*^R ^lacking the 5' LoxP site, we used a short (0.5 kb) left arm upstream of the 5' LoxP site. This was used to ensure that during homologous recombination between the left arm and the endogenous sequence, the crossing over could take place not only upstream of the 5' LoxP site, leading to its incorporation into the endogenous gene, but also downstream of the LoxP site, preventing its incorporation. The targeted allele retaining the 5' LoxP site (*Baf57^Int^*) can be converted to *Baf57^F ^*after FLP-mediated excision of the gene-trap cassette from the germline, whereas the allele lacking the 5' LoxP site is Cre-resistant and acts as *Baf57^ΔR^*.

**Figure 3:**
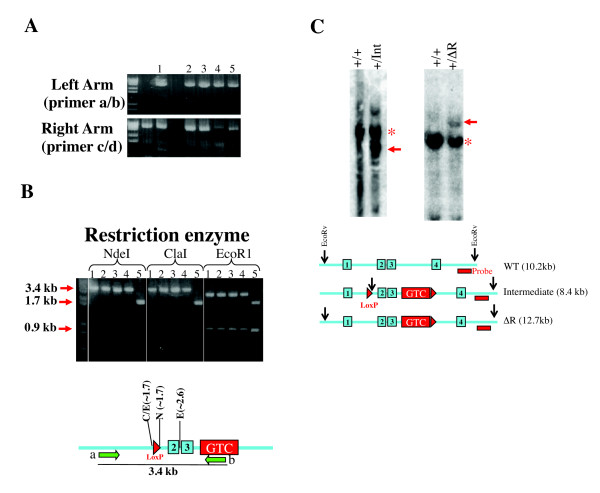
**Identification of embryonic stem (ES) clones bearing *Baf57^Int ^*and *Baf57^ΔR^***. (**A**) PCR screening for ES clones with correctly integrated arms. We first used the primer pair a/b (Figure 2) to screen for left-arm integration. Of twenty clones examined, seven gave positive results; five of these seven clones were confirmed by re-screening (top), and in each of the five clones, the right arm was correctly integrated based on PCR using primer pair c/d depicted in Figure 2 (bottom). (**B**) To determine if the 5' LoxP site is present in these five clones, we digested the PCR products amplified with the primer set a/b with restriction enzymes recognizing cloning sites at the LoxP site, which showed that clone 5 carried the 5' Lox P site and hence the *Baf57^Int^*. Of note, an additional *Eco*R1 site was present within the gene-trapping cassette, hence there were cleavages in all five amplicons. The 3.4 kb amplicon (Figure 2) is depicted at the bottom, with the approximate positions (in kb) of the restriction sizes indicated. The green arrows denote PCR primers as in Figure 2. C, *Cla*I, E, *Eco*R1, N, *Nde*I. (**C**) Southern blotting confirmed the identities of *Baf57^Int ^*and *Baf57^ΔR^*. The asterisks and arrows indicate the genomic fragments released (by EcoRV digestion) from the WT and the targeted alleles, respectively. The strategy of the assay is diagrammed at the bottom.

We recovered both *Baf57^Int ^*and *Baf57^Δ^*^R ^after screening only 20 ES cell clones, confirming that the targeting method, called 'targeted trapping' [38], is extremely efficient (Figure 3). Of note, only one of the five correctly targeted clones carried the 5' LoxP site; this frequency could perhaps be increased by lengthening the 0.5 kb left arm upstream of the LoxP site. We then generated *Baf57^Int/+ ^*and *Baf57^ΔR/+ ^*mice using standard methods. The heterozygous pups were born at normal Mendelian ratios, but homozygous pups were absent, indicating embryonic lethality. Both strains widely expressed GFP, with the GFP expression in T cells in *Baf57^Int/+ ^*mice abolishable by *CD4-Cre*, indicating that *Baf57^Int ^*is useful for reporting Cre activity (see Additional file 1, Figure S1). Finally, we generated *Baf57^F ^*by deleting the gene-trap cassette in *Baf57^Int ^*using a line ubiquitously expressing FLPe.

### Characterization of *Baf57^F ^*and *Baf57^ΔR^*

**Figure 4:**
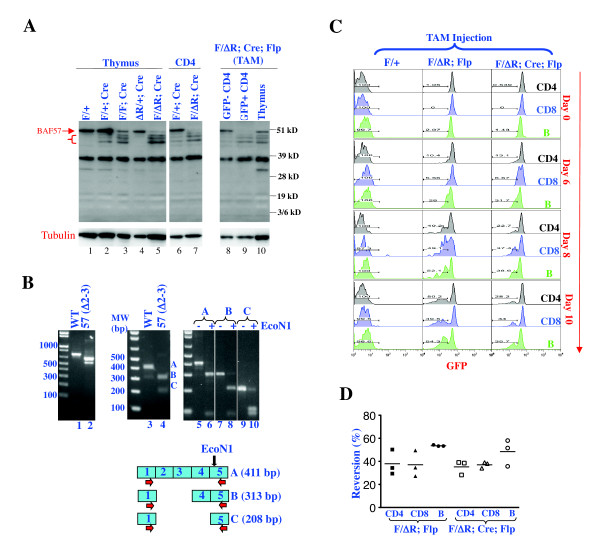
**Behaviors of *Baf57^Int ^*and *Baf57^ΔR^***. (**A**) Western blot examining Brg/Brahma-associated factor (BAF)57 expression in total thymocytes (lanes 1 to 5, and lane 10) and purified CD4 cells (lanes 6 to 9). Samples in lanes 8 to 10 were from mice exposed to TAM. The genotype of the mice are indicated, where F, ΔR and Flp denote *Baf57^F^, Baf57^ΔR ^*and *R26^FlpoER1^*, respectively. The bracket at the left indicates the three BAF57 mutant proteins expressed from *Baf57^F ^*after deletion of floxed sequence. At least three mice of each genotype were analyzed. Shown is a representative experiment. (**B**) Reverse transcriptase (RT)-PCR examining BAF57 transcripts in CD4 cells expressed by the wild-type (WT) allele (lanes 1 and 3) or the mutant allele lacking exons 2 and 3 (lanes 2 and 4); the cells were isolated from *Baf57^+/ΔR^; CD4-Cre; R26^FlpoER1 ^*and *Baf57^F/ΔR^; CD4-Cre; R26^FlpoER1 ^*mice, respectively. RT-PCR primers targeted exons 1 and 7 (lanes 1 and 2) or 1 and 5 (lanes 3 and 4). The latter primer set yielded amplicons A to C (lanes 3 and 4, diagrammed at the bottom), which were re- amplified with a nested primer set, gel-purified, and digested with *Eco*N1 to verify their identities (lanes 5 to 10). There was also a nonspecific amplicon (about 310 bp, lane 3) which was not re-amplifiable (not shown). Two mice of each genotype were analyzed. Shown is a representative experiment. (**C**). Flow cytometric assays monitoring green fluorescent protein (GFP) expression in peripheral blood CD4, CD8 and B cells at various times after tamoxifen (TAM) injection. Three mice of each genotype were analyzed. (**D**) Summary of the reversion efficiencies at day 10 in CD4, CD8, and B cells, as measured by the fraction of cells that had lost GFP. Each symbol represents an individual mouse.

We first examined *Baf57^F/+^; CD4-Cre *mice carrying the *CD4-Cre *transgene that directs Cre expression in CD4+ T cells (i.e., DP and CD4 cells) [39]. PCR analysis confirmed that the floxed sequence was effectively deleted from thymocytes (not shown). To determine the status of the BAF57 protein, we performed western blotting using an antibody against the C-terminus of BAF57. In both the thymocytes and CD4 cells from *Baf57^F/+^; CD4-Cre *mice, Cre-mediated deletion led to the emergence of three truncation mutants of BAF57 that were much less abundant than WT BAF57 (Figure 4A, lanes 2 and 6). The mutants seemed upregulated in *Baf57^F/F^; CD4-Cre *thymocytes where both copies of *Baf57^F ^*were subject to deletion (Figure 4A, lane 3). Of note, a low level of WT BAF57 persisted in total thymocytes from these mice, which presumably came from the DN subset lacking Cre expression. We next examined *Baf57^ΔR^*, alone and in combination with *Baf57^F^*. As expected, *Baf57^ΔR ^*did not express a mutant protein detectable by the antibody (Figure 4A, lane 4), whereas the *Baf57^F/ΔR^; CD4-Cre *thymocytes harbored the three truncation mutants, as well as a trace amount of WT BAF57 that was less abundant than in *Baf57^F/F^; CD4-Cre *mice (as expected from the fact that the latter carried two copies of *Baf57^F ^*(Figure 4A, lane 5). Finally, mature CD4 cells from *Baf57^F/ΔR^; CD4-Cre *mice also expressed the three truncation mutants, but WT BAF57 was absent in these cells, indicating complete deletion of the floxed allele (Figure 4A, lane 7). There was no gross defect in the development or function of T cells in any of the mice described above (not shown).

To determine whether *Baf57^ΔR ^*could be conditionally activated through deletion of the gene-trap cassette, we used the *R26^FlpoER1 ^*deleter strain that ubiquitously expresses FlpoER1 from the Rosa26 locus [40]. FlpoER1 is a fusion between the codon-optimized FLP called FLPo [41,42] and the modified estrogen receptor (ER), ER^T2^, which retains the recombinase in the cytosol until tamoxifen (TAM) administration [43], with a linker sequence derived from Cre-ER inserted between the FLPo and ER^T2^. In mice carrying *Baf57^ΔR ^*and expressing FlpoER1, TAM injection therefore would induce nuclear translocation of FlpoER1 to cause deletion of the gene-trap cassette and hence activation of the *Baf57^ΔR^*. To determine the ability of FlpoER1 to activate *Baf57^ΔR^*, we introduced *R26^FlpoER1 ^*into *Baf57^F/ΔR ^*mice. We injected TAM once a day for 3 consecutive days, and monitored GFP expression in peripheral blood lymphocytes. Before TAM injection, GFP was uniformly expressed in CD4, CD8 and B cells (Figure 4C, top middle panel). Cells losing GFP emerged 6 days after the first TAM injection, and comprised around 50% of total lymphocytes on day 10, when the GFP signals in the affected cells were reduced by about 4-fold (Figure 4C, bottom middle plot). Because the GFP half-life is ~24 hours and the peripheral lymphocytes are mostly resting, deletion of the gene-trap cassette in our mice seemed to occur predominantly around day 8 after the first TAM injection. A similar observation was made in a *Baf57^F/ΔR ^; CD4-Cre; R26^FlpoER1 ^*mouse, except that on day 10, only about 30% of the lymphocytes had deleted the gene-trap cassette (Figure 4C, right). The variation in the deletion efficiency was stochastic and not correlated with genotype; on average, the deletion efficiencies at day 10 were 37 ± 8%, 37 ± 7%, and 51 ± 8% in CD4, CD8, and B lymphocytes, respectively (Figure 4D). We sorted GFP^+ ^and GFP^low/- ^cells from *Baf57^F/ΔR^; CD4-Cre; R26^FlpoER1 ^*mice, and performed western blotting. As expected, the GFP^+ ^cells expressed only the three truncation mutants and no WT BAF57 protein (Figure 4A, lane 9), and importantly, WT BAF57 was restored in GFP^low/- ^cells, showing that *Baf57^ΔR ^*can indeed be converted into the WT allele (Figure 4A, lane 8).

We next sought to determine the identities of the three mutants expressed by *Baf57^F ^*after Cre-mediated deletion of exons 2 to 3 (these mutant referred to as *Baf57^Δ(2-3) ^*hereafter). As mentioned before, *Baf57^Δ(2-3) ^*is expected to direct the expression of the BAF57 mutant that lacks the first 18 residues and hence is about 2 kDa smaller than the WT protein, which might account for one of these three mutants, as the molecular weights of these mutants seemed to differ from the WT protein by less than 5 kDa. The other two mutants might be its degradation products, and/or be expressed from aberrantly spliced transcripts. To address this, we performed reverse transcriptase (RT)-PCR using primers targeting exons 1 and 7. The primers amplified a single product of 839 bp in WT CD4 cells as expected, but produced two smaller bands from *Baf57^F/ΔR ^; CD4-Cre *mice (Figure 4B, lanes 1 and 2). RT-PCR using primers targeting exons 1 and 5 suggested that the top and bottom bands in the mutant cells represented the predicted transcript (with exon 1 joined to exon 4) and an aberrant transcript with exon 1 joined to exon 5, respectively (Figure 4B, lanes 3 and 4), which was confirmed by restriction enzyme digestion of the amplicons (lanes 5 to 10). Interestingly, exon 5 was also found to harbor an in-frame ATG, suggesting that the aberrantly spliced transcript can be translated into a deletion mutant lacking the N-terminal 70 aa (including the first 5 aa of the HMG domain) and hence is about 8 kDa smaller than WT protein. Perhaps this mutant was running aberrantly slowly to constitute one of three mutant bands. Of note, as in the case of the ATG in exon 4, the ATG in exon 5 is not embedded in the Kozak consensus sequence, consistent with their low expression levels compared with the WT protein. Thus, at least two of the three mutant proteins might result from translation of spliced transcripts. Indeed, multiple alternatively spliced transcripts, two of them predicted to direct the expression of the mutant proteins lacking the N-terminal 18 and 70 aa, normally exist in the brain [44]. However, direct sequencing of these bands is needed to confirm this hypothesis, particularly because of the unusual mobility of BAF57, whose predicted molecular weight is 45 kDa but whose apparent molecular weight ranges from 50 to 57 kDa, depending on the gel system used.

Finally, as alluded to before, although the three mutant proteins were expressed, as expected, only at very low levels in the cells that also expressed WT protein, they accumulated in the cells lacking the WT protein, which occurred both in the thymus (Figure 4A, lanes 2 versus 5) and the mature CD4 cells (Figure 4A, lanes 6 versus 7, and 8 versus 9). This upregulation of the mutant proteins in the absence of WT BAF57 presumably reflected a post-translational regulatory mechanism seeking to maintain stoichiometric abundance of various BAF subunits [45]. As the mutant proteins are at least partially active, their accumulation may help explain why deletion of the floxed exons in *Baf57^F ^*had no major biological effect. However, because the mutants presumably lacked the intact N-terminus, their accumulation might recapitulate, to some extent, the phenotype seen in mice overexpressing a BAF57 dominant-negative mutant lacking the N-terminal 133 aa, and more importantly, this defect may be prevented by TAM treatment. This is indeed the case, as described below.

### Phenotype caused by *Baf57 *mutations and its prevention by TAM injection

**Figure 5:**
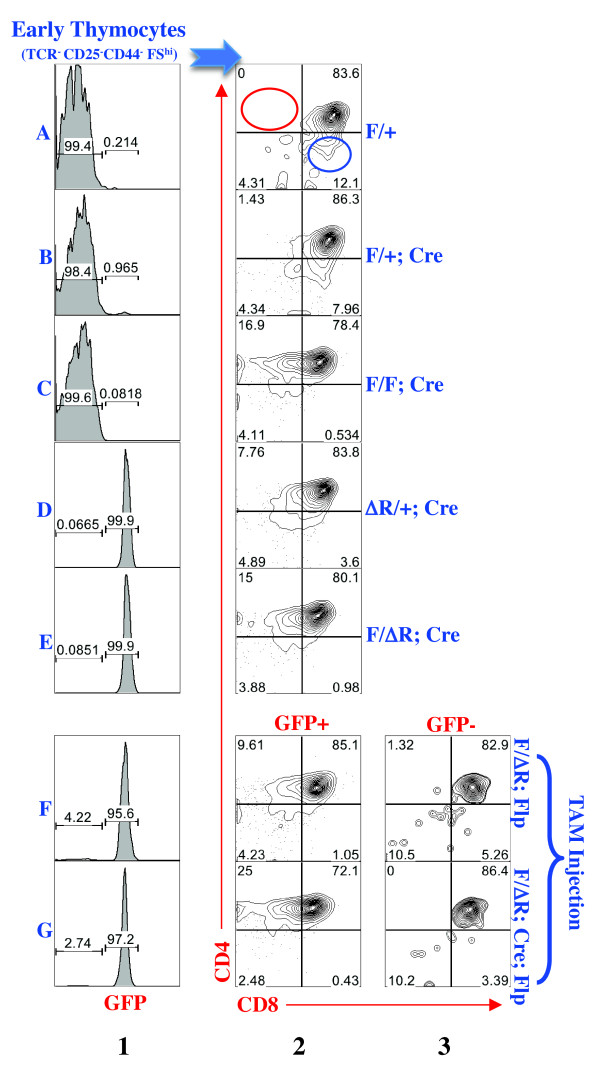
***Baf57 *mutations impaired CD8 expression, which was preventable by tamoxifen (TAM)**. Thymocytes were stained with T-cell receptor (TCR) CD4, CD8, CD25 and CD44 antibodies. The early thymocyte population, which are large cells (defined by high forward scatter or FS^hi^) that lack TCR, CD25 or CD44, were resolved into GFP^- ^and GFP^+ ^subsets (column 1) before analysis of CD4/CD8 expression (columns 2 to 3). The genotype symbols are as described in Figure 4A. Of note, a CD4^- ^CD8^+ ^population, which made up 12% of the early thymocytes in WT mice (blue circle in row A, column 2), was absent in *Baf57^F/F^; CD4-Cre *mice, and partially depleted by *Baf57^ΔR^*, with unknown mechanisms. At least three mice of each genotype were analyzed. Shown is a representative experiment.

A major defect caused by overexpressing the BAF57 mutant lacking the N-terminal 133 aa is the dramatically impaired CD8 expression in early DP cells, which is found in the large thymocyte blasts lacking T-cell receptor (TCR), CD25 or CD44 expression (designated TCR^-^CD25^-^CD44^-^FS^hi^) [21,24,34]. In control mice, this early thymocyte population comprised mostly (84%) DP cells and no CD4^+^CD8^low/- ^cells, the latter being the hallmark of impaired CD8 expression (Figure 5, row A, column 2, where the red circle denotes the absence of CD4^+^CD8^low/- ^cells). Heterozygous deletion of the floxed exons in *Baf57 *had little effect (row B), whereas homozygous deletion (in *Baf57^F/F^; CD4-Cre *mice) led to significant accumulation of CD4^+^CD8^low/- ^cells, which made up 17% of the early thymocyte population (Figure 5, row C), although the effect was two-fold weaker than in previously reported BAF57 dominant-negative mice containing 34% of the CD4^+^CD8^low/- ^cells [24]. Interestingly, a single copy of *Baf57^ΔR ^*was sufficient to cause a mild defect in CD8 expression (Figure 5, row D) whereas a single copy of *Baf57^Δ(2-3) ^*was not (Figure 5, row B), despite the fact that *Baf57^Δ(2-3) ^*was expressed at very low levels if at all (Figure 4A, lane 2). The cause of this discrepancy is unclear but we have excluded GFP expression in the former mice as a potential mechanism (see Additional file 1, Figure S2). Finally, as expected, *Baf57^F/ΔR^; CD4-Cre *mice also had a significant defect in CD8 expression (Figure 5, row E). Another major defect caused by overexpressing the BAF57 mutant lacking the N-terminal 133 aa is premature CD4 de-repression in DN cells [24,33]. No such defect was seen in *Baf57^F/F^; CD4-Cre *mice, as predicted from the Cre expression pattern, and neither could *Baf57^ΔR ^*cause this defect, indicating that BAF57 is haplosufficient for CD4 repression in DN cells (not shown).

The fact that *Baf57^F/ΔR^; CD4-Cre *mice showed a significant defect in CD8 expression set the stage for testing the effect of TAM on the phenotype. Given the rapidity of early T-cell development and the relatively slow kinetics of *Baf57^ΔR ^*activation, it is unfeasible for TAM to 'cure' the pre-existing phenotype in early DP cells that exist only transiently, but TAM might be able to activate *Baf57^ΔR ^*in the precursors of these transient cells to prevent the subsequent defective CD8 expression in early DP cells. To test this, we used *Baf57^F/ΔR^; CD4-Cre; R26^FlpoER1 ^*and *Baf57^F/ΔR^; R26^FlpoER1 ^*mice, the latter included because *Baf57^ΔR ^*alone sufficed to produce a detectable, albeit weak, defect in CD8 expression. The mice were exposed to TAM as described above. Unexpectedly, on day 10 after the initial TAM injection, only about 5% of the early thymocytes had lost GFP expression (Figure 5, rows F to G, column 1), and the same was true for later thymocytes (not shown), in contrast to the approximately 37% deletion efficiency in peripheral lymphocytes, thus revealing substantial tissue-specificity in the deletion efficiency in our system. We then examined early DP cells showing or lacking GFP expression. As expected, in cells expressing GFP, CD8 expression was impaired, with the CD4^+^CD8^low/- ^population comprising about 10% and 25% of the early thymocytes in *Baf57^F/^; R26^FlpoER1^*and *Baf57^F/ΔR^; CD4-Cre; R26^FlpoER1 ^*mice, respectively (Figure 5, rows F to G, columns 2). Importantly, the CD4^+^CD8^low/- ^population was absent in the GFP^- ^compartment, demonstrating successful prevention of the phenotype (Figure 5, rows F to G, column 3). This effect was dependent on *R26^FlpoER1 ^*(not shown) and thus not an artifact resulting from elimination of GFP (see Additional file 1, Figure S2).

## Discussion

### Strengths and limitations of LOFT

LOFT combines pre-existing basic genetic methods into a straightforward and reliable reversible gene-targeting method. The method is reliable because its two components, Cre-mediated conditional gene targeting and FLP-mediated reversible gene trapping, are both well established. It is also simple because the pair of alleles involved can typically be generated with a single construct. Furthermore, for the genes whose floxed alleles are already available, only the trapped alleles are needed to convert the pre-existing cKO into reversible cKO, which simplifies the method. This is an important advantage because floxed alleles for the majority of mouse genes will become available in the future thanks to concerted efforts in several countries [46,47]. LOFT is also flexible, because the two alleles can be used independently for conventional gene targeting, and what is more, the 'intermediate allele' generated can serve as a GFP reporter of Cre activities. LOFT does require Cre and FLP-deleter lines, but this should not pose a problem because numerous Cre lines are already available, as is a mouse line ubiquitously expressing a version of FlpoER that is far more effective than the one (FlpoER1) used in the current study; the two versions are identical except for the linker sequence between FLPo and ER [40]. Finally, LOFT, whose major application is likely to be reversible cKO, can have other applications such as reversal of hypomorphic alleles, as we have shown. As another application, point mutations may be introduced into the trapped genes to dissect their functions, which is analogous to the approach for producing conditional point mutant mice that we previously developed [21], except that in the previous method, the point mutant is expressed concurrently with the loss of the WT protein, whereas in the LOFT method, the two events can happen sequentially. The sequential occurrence would be essential in addressing, for example,, the mode of action of P53. Specifically, KO of the P53 gene is known to cause tumors, which can be suppressed by restoration of P53 expression [1,2]. Surprising, it was recently found that a P53 point mutant unable to induce apoptosis, cell-cycle arrest, or senescence retained the ability to prevent tumorigenesis, presumably as a result of the ability of the mutant protein to regulate energy metabolism and production of reactive oxygen species [48]. Whether the mutant can also suppress pre-existing tumors is unclear, and this important question is readily addressable by expressing the mutant protein in P53 cKO mice. In summary, LOFT is a straightforward, reliable, simple, and flexible method for both reversible and conventional (constitutive or conditional) gene targeting, and is readily adaptable for other applications.

However, there are several limitations to LOFT. First, the method involves a pair of alleles with the corresponding pair of recombinases, thus entailing significant amounts of breeding. Second, the trapped allele is null by default, and so the gene needs to be haplosufficient for mouse survival. Third, the method is not suitable for reversing the effect of deletion of regulatory elements such as enhancers or silencers. Fourth, because LOFT works by combining Cre/Lox and gene-trapping systems, any limitation in these basic genetic methods would apply to LOFT. For example, as mentioned above, if the target gene is not expressed in ES cells, then 'targeted trapping' is not applicable, and conventional methods, which have much lower efficiency, must be used [38]. Fortunately, over 65% of all protein-coding genes in the mouse genome are amenable for promoter trapping in ES cells [49], and the efficiency can be raised to 85% if the binding sites (which can be made removable) for a transcription factor expressed in ES cells (Oct4) are engineered into the vector [50]. Thus, inefficient conventional methods should be reserved for only around 15 to 35% of protein-coding genes. Because conventional methods are well established, we do not expect unusual problems in their application to our setting. Another example of the limitation of LOFT is that if two targeting constructs are needed to insert the 5' and 3' LoxP sites, as in the case of floxing a large DNA fragment, then creating the allelic series in LOFT will accordingly require two constructs. Furthermore, Cre/FLPo cannot always efficiently delete target sequences. Indeed, FLPo-catalyzed removal of the gene-trap cassette at the BAF57 locus was only around 5% in the thymus by day 10. This problem can be addressed by monitoring the deletion via GFP expression, and by the use of the new version of conditional FLP ("FlpoER") that is efficient 'in any tissue at any time during development or in the adult' [40]. With this version of FLP, Joyner and colleagues found that a single injection of TAM was sufficient to induce widespread and efficient deletion of a reporter gene in the embryos and adults within 4 and 7 days, respectively, whereas the version used in the current study (FlpoER1) barely works under this condition [40]. The new enzyme is expected to make LOFT widely applicable. However, the old version (FlpoER1), which is efficient in peripheral T cells but not thymocytes, has a unique advantage for studying developmental programming of T-cell functions. As mentioned above, such studies entail gene inactivation in the thymocytes and subsequent reactivation in mature T cells. As the thymus continues to export T cells into the periphery in adults, the peripheral T-cell pool would be significantly contaminated with the confounding T cells that have undergone premature reactivation, if FLP is allowed to work efficiently in the thymocytes. The final limitation of LOFT involves the fact that most of the floxed alleles recently generated by the European Conditional Mouse Mutagenesis (EUCOMM) carry an FRT site outside the floxed sequence. If these alleles are paired with reversible KO alleles, interchromosomal recombination can occur after FLP activation. These recombination events are presumably too rare to confound data interpretation, unless they lead to dominant effects such as tumorigenesis. However, such effects are in themselves interesting, and so the nuisance may be a blessing in disguise.

### Utility of *Baf57^ΔR ^*and *Baf57^F^*

BAF57^ΔR ^is a null allele that can be rescued by deleting the gene-trap cassette. BAF57^ΔR ^homozygous mice are apparently embryonic lethal, which precludes the analysis of the effect of BAF57 KO in adult tissues. This problem may be solved by deleting the gene-trap cassette (and hence restoring BAF57 expression) in a fraction of cells in the embryo, which may rescue the embryo to produce mosaic adults containing BAF57 KO, Het, and WT cells, which are distinguishable based on (the level of) GFP expression. Such mosaic mice will be the source of cells lacking BAF57.

In contrast to *Baf57^ΔR^*, *Baf57^F ^*was designed to be a conditional hypomorphic allele expressing a deletion mutant at a low level, following excision of the floxed sequence. Instead, we detected three truncated proteins that seemed to result, at least in part, from alternatively spliced transcripts. In addition, although the mutants were indeed expressed at very low levels in the presence of the allele expressing WT BAF57, they accumulated in the absence of WT BAF57. Despite these unexpected changes, homozygous deletion of the floxed exons in DP cells caused a phenotype resembling, albeit weaker than, that resulting from overexpressing the BAF57 dominant-negative mutant lacking the first 133 aa, confirming that *Baf57^F ^*is a conditional hypomorphic allele. We are now extending the analysis to other tissues, by deleting the floxed exons from the germline. We suspect that the mice lacking the two exons will be viable, but may display some specific defects. This allele may thus enable us to interrogate the role of BAF57 in a way not feasible with any BAF57 null allele, whether the null is constitutive or conditional. Finally, our ultimate goal of developing this method is to study the potential role of BAF57 in epigenetic programming of mature T cells. Although no gross functional defects in mature T cells were detected in *Baf57^F/ΔR^; CD4-Cre; R26^FlpoER1 ^*mice, some specific, subtle defects may exist. Because the mature T cells are resting, it would be possible to test whether restoring BAF57 expression in these cells can rescue the pre-existing phenotype, and we are therefore systematically searching for the putative functional defects in the mature T cells.

## Conclusions

Reversible regulation of endogenous genes in mice is necessary for addressing multiple important biological questions. We have combined the Cre/Lox and gene-trap systems to develop LOFT, a reliable and straightforward reversible cKO method. LOFT lacks the limitations of the pre-existing reversible gene regulatory systems, and can also be used to producing traditional constitutive KO and cKO mice. It offers an advantageous alternative to the conventional gene-targeting methods.

## Methods

### DNA construct, embryonic stem cell targeting, and mouse breeding

The targeting construct (depicted in Figure 2) was based on the pEZ FRT Lox backbone (a gift of K. Rajewsky). This plasmid carries the FRT-flanked promoter-Neo expression cassette, which is in turn flanked by LoxP sites. The FRT-flanked cassette was replaced by a synthetic fragment bearing an FRT-flanked splicing acceptor together with multiple cloning sites. A Neo-Ires-GFP-SV40 polyA fragment was inserted immediately downstream of the splicing acceptor to create the gene-trapping cassette. The left and right arms flanking the cassette, encompassing BAF57 exons 2 to 3 and exon 4, respectively, were amplified by PCR from C57B/6 mice. The cloned DNA was sequenced in its entirety, and no PCR-introduced error was found. The construct was electroporated into 129/sv ES cells, and corrected targeted clones were identified by PCR and Southern blots. The *CD4Cre *transgenic mice (on C57B/6 background, a gift of C. Wilson) have been described previously [39]. The effects of the BAF57 dominant-negative mutation or Brg1 KO on T cell development are robust and observable on mixed genetic backgrounds [21,24,34]. *R26^FlpoER1 ^*mice (on C57B/6 background) were a gift from A. L. Joyner [40]. The primers for screening the ES cells are shown in Table 1.

**Table 1:** Primers used in experiments

Purpose		Sequence (5' → 3')
Screening ES cells	Left arm F	CCGCCTACATTCTCCATCTTCTCCA

	Left arm R	CAGTCCCTTCCCGCTTCAGTGACAA

	Right arm F	TACGTATGGCACATAGAACTTGATA

	Right arm R	GAGCACCCAGTCCGCCCTGAGCAAA

RT-PCR	Forward	CGGGACAAAGGGAAGCGAAG

	Reverse	CGCCACATGCCACCAATAATC

Nested PCR for amplicons produced by RT-PCR	Forward	GGACAAAGGGAAGCGAAGCCGGAGCTG

	Reverse	GCCACATGCCACCAATAATCTTGCCAAT

Production of probe for Southern blot	Forward	CCACTCCCCGTGGAACACGC

	Reverse	CCGTGACCCGGCTGTTGGTG

Abbreviations: ES, embryonic stem; RT, reverse transcriptase.

### Reverse transcriptase PCR

Total RNA was isolated from sorted CD4 cells with RNAeasy plus (Qiagen) and amplified by a one-step RT-PCR kit (Qiagen Inc., Valencia, CA, USA). The amplicons were re-amplified by nested PCR, gel-purified, and digested with *Eco*N1.

### Southern and western blotting

Genomic DNA (10 ug) was digested with *Eco*RV and run on a 0.8% agarose gel. The probe was a 1.5 kb PCR product amplified with the primer pair shown in Table 1.

For western blotting, 0.3 million cells were run on a gel (NuPAGE^® ^Novex 4-12% Bis-Tris gel; Invitrogen Corp., Carlsbad, CA, USA) in MOPS running buffer using a commercial protein standard (BenchMark™ Prestained Protein Standard; Invitrogen) as the molecular weight marker. The membrane was probed with a BAF57 antibody directed against the C-terminus of BAF57, before re-probing with an anti-tubulin antibody as loading control. The primary antibodies were detected using horseradish peroxidase-conjugated secondary antibodies, which were visualized with enhanced chemiluminescence reagents on radiography films.

### Tamoxifen injection and flow cytometric analysis

TAM solution (20 mg/ml) was prepared by dissolving 200 mg TAM (free base; T5648; Sigma-Aldrich, St Louis, MO, USA) to 0.5 ml ethanol before adding 9.5 ml autoclaved peanut oil. The solution was sonicated and stored at -20°C. To delete the gene-trap cassette, 100 ul of the solution was injected intraperitoneally into adult *BAF57^F/ΔR^; Cre; R26^R26FlpoER1 ^*mice once a day for 3 consecutive days. To monitor the effect of TAM, a few drops of peripheral blood were treated with red blood cell lysis buffer, and the cells were then stained with anti-CD4-APC, anti-CD8-PE-Cy7, and anti-B220-PE before flow cytometric analysis of GFP expression in lymphocytes. To determine the effects of BAF57 mutation on early T-cell development, thymocytes were stained with anti-CD4-APC, anti-CD8-PE-Cy7, anti-CD25-PE, anti-CD44-FITC and anti-CD3-Pacific blue, and the data, collected on the flow cytometers (LSRII; BD Biosciences Inc., San Jose, CA, USA), were analyzed as described previously [21,24,34].

## Competing interests

The authors declare that they have no competing interests.

## Authors' contributions

BC, RK, JZ, and JW performed experiments; RF helped generate the mice; and TC designed the project and wrote the manuscript. All authors have read and approved the manuscript for publication.

## Supplementary Material

Additional file 1 **Supplemental Figures**. Figure S1. *Baf57^int ^*served as reporter of Cre activity. Peripheral blood lymphocytes were analyzed for green fluorescent protein (GFP) expression. The genotypes of the mice are indicated at the top, where Cre represents the *CD4Cre *transgene expressing Cre in the double-positive (DP) cells during T-cell development. CD4 and CD8 cells are progeny of DP cells, and thus had undergone Cre-mediated excision at *Baf57^int^*. Figure S2. GFP expression did not impair CD8 expression in early double-positive (DP) cells. Thymocytes from WT mice or transgenic mice carrying a transgene with wide expression of green fluorescent protein (GFP) were stained with CD4, CD8, CD25, and CD44 antibodies, and the cells were analyzed essentially as described previously [21,24,34]. Thymocytes consist of cells at various stages of development. Among the cells at the earliest stages were those that lacked T-cell receptor (TCR) expression and are large in size, as marked by the gate in column 1. The cells in this gate were mostly CD25^-^CD44^- ^(column 2). These CD25^-^CD44^- ^cells, GFP^- ^in WT mice but GFP^+ ^in transgenic mice as expected (column 3), consisted mostly of early DP cells (column 4). CD8 expression in these early DP cells were impaired by Brg/Brahma-associated factor (BAF)57 mutations, but not by GFP expression. Click here for file
