# Supplementary figures and images for: LoxP-FRT Trap (LOFT): a simple and flexible system for conventional and reversible gene targeting
Source: BMC Biol. 2012 Nov 30;10:96. doi: 10.1186/1741-7007-10-96 (PMC3529186; doi:10.1186/1741-7007-10-96)

**Fig. S1**

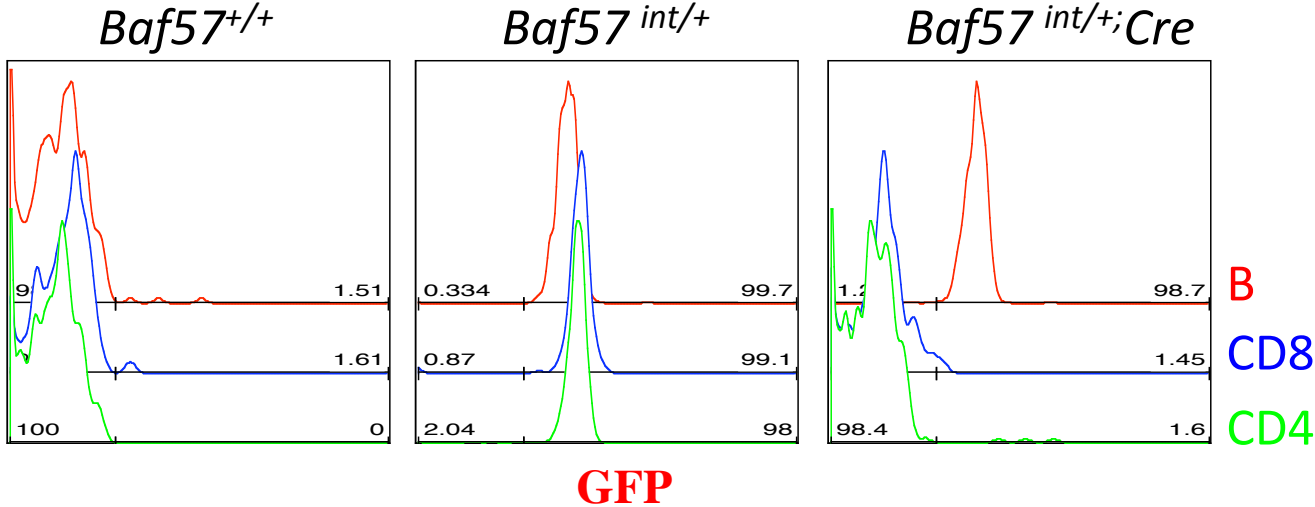

Fig. S2

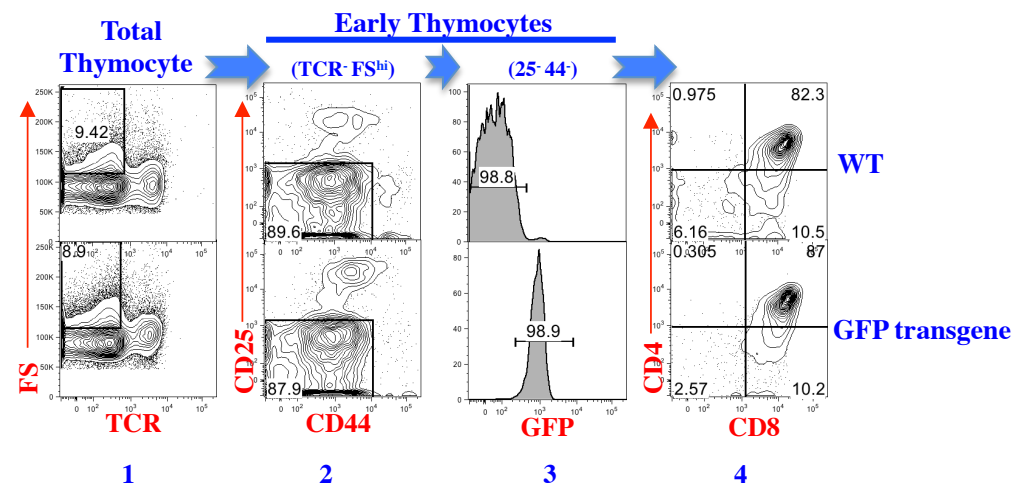

Supplement: Additional file 1 — Supplemental Figures. Figure S1. Baf57int served as reporter of Cre activity. Peripheral blood lymphocytes were analyzed for green fluorescent protein (GFP) expression. The genotypes of the mice are indicated at the top, where Cre represents the CD4Cre transgene expressing Cre in the double-positive (DP) cells during T-cell development. CD4 and CD8 cells are progeny of DP cells, and thus had undergone Cre-mediated excision at Baf57int. Figure S2. GFP expression did not impair CD8 expression in early double-positive (DP) cells. Thymocytes from WT mice or transgenic mice carrying a transgene with wide expression of green fluorescent protein (GFP) were stained with CD4, CD8, CD25, and CD44 antibodies, and the cells were analyzed essentially as described previously [21,24,34]. Thymocytes consist of cells at various stages of development. Among the cells at the earliest stages were those that lacked T-cell receptor (TCR) expression and are large in size, as marked by the gate in column 1. The cells in this gate were mostly CD25-CD44- (column 2). These CD25-CD44- cells, GFP- in WT mice but GFP+ in transgenic mice as expected (column 3), consisted mostly of early DP cells (column 4). CD8 expression in these early DP cells were impaired by Brg/Brahma-associated factor (BAF)57 mutations, but not by GFP expression. [file 1741-7007-10-96-S1.PDF]
